# The “Brittle Response” to Parkinson’s Disease Medications: Characterization and Response to Deep Brain Stimulation

**DOI:** 10.1371/journal.pone.0094856

**Published:** 2014-04-14

**Authors:** Daniel Martinez-Ramirez, Juan Giugni, Vinata Vedam-Mai, Aparna Wagle Shukla, Irene A. Malaty, Nikolaus R. McFarland, Ramon L. Rodriguez, Kelly D. Foote, Michael S. Okun

**Affiliations:** 1 Department of Neurology, Center for Movement Disorders and Neurorestoration, University of Florida, Gainesville, Florida, United States of America; 2 Department of Neurosurgery, Center for Movement Disorders and Neurorestoration, University of Florida, Gainesville, Florida, United States of America; University of Toronto, Canada

## Abstract

**Objective:**

Formulate a definition and describe the clinical characteristics of PD patients with a “brittle response” (BR) to medications versus a “non-brittle response” (NBR), and characterize the use of DBS for this population.

**Methods:**

An UF IRB approved protocol used a retrospective chart review of 400 consecutive PD patients presenting to the UF Center for Movement Disorders and Neurorestoration. Patient records were anonymized and de-identified prior to analysis. SPSS statistics were used to analyze data.

**Results:**

Of 345 included patients, 19 (5.5%) met criteria for BR PD. The BR group was comprised of 58% females, compared to 29% in the NBR group (*P* = .008). The former had a mean age of 63.4 compared to 68.1 in the latter. BR patients had lower mean weight (63.5 vs. 79.6, *P = <*.001), longer mean disease duration (12.6 vs. 8.9 years, *P* = .003), and had been on LD for more years compared to NBR patients (9.8 vs. 5.9, *P* = .001). UPDRS motor scores were higher (40.4 vs. 30.0, *P* = .001) in BR patients. No differences were observed regarding the Schwab and England scale, PDQ-39, and BDI-II. Sixty-three percent of the BR group had undergone DBS surgery compared to 18% (*P* = .001). Dyskinesias were more common, severe, and more often painful (*P = <*.001) in the BR group. There was an overall positive benefit from DBS.

**Conclusion:**

BR PD occurred more commonly in female patients with a low body weight. Patients with longer disease duration and longer duration of LD therapy were also at risk. The BR group responded well to DBS.

## Introduction

Recently, a particular group of Parkinson’s disease (PD) patients was reported to be at higher risk of levodopa-induced dyskinesia; young female patients with low body weight [1]. These patients were more sensitive to regular treatments. The term “brittle” has been used in medicine to describe an unstable condition in diabetics with poor glycemic control [2], leading to more expensive care and to a risk for severe complications. The expression “brittle” with reference to PD was first employed in 1982 to describe patients who seemed susceptible to adverse effects when treated with small amounts of levodopa (LD) [3]. In addition to dyskinesias, some patients with a BR may spend only a tiny portion of their day in the optimal “on” medication state. Hence, for these patients, the prescribed medication regimen can be complicated, and the therapeutic window narrow. The approach to these patients in clinical practice has been to administer smaller doses of PD medications more frequently throughout the course of a day, however there is no standardized or well-accepted approach beyond expert opinion.

Because the “brittle” phenomenon has not been described in a large cohort of patients, we sought to formulate a definition, and to describe the clinical characteristics of a “brittle response” (BR) group vs. a “non-brittle response” (NBR) control group. We also sought to better characterize the use of deep brain stimulation (DBS) for this population.

## Methods

The University of Florida Institutional Review Board approved the study. A retrospective chart review of 400 consecutively seen PD patients over a period of 6 months was conducted at the University of Florida Center for Movement Disorders and Neurorestoration. Patient records were anonymized and de-identified prior to analysis. BR patients were defined as taking 100 mg or less of levodopa per dose and were required to report symptoms of disabling dyskinesia(s), in their most recent visit or in their last visit before having DBS surgery. Our definition only includes patients who cannot tolerate a maximal dose for any administration during the day; if they can tolerate a higher alternating dose they would be eliminated as a BR patient. Basic demographic information, current treatment doses, Unified Parkinson’s Disease Rating Scale (UPDRS) scores in the “on” medication state, Hoehn-Yahr (HY) stage, Schwab and England scale (SE), Parkinson’s Disease Questionnaire (PDQ-39), and Beck Depression Inventory (BDI-II) scores were all extracted from each patient’s chart, as well as from an Institutional Review Board (IRB) approved database (INFORM-PD). In all scales, except for SE, higher scores indicate more or worse symptoms. Severity of dyskinesia was measured according to its grade of disability using the UPDRS part IV item 33. Clinical subtype of PD was classified according to type of presentation [4]. Fifty-five patients were excluded from the final analysis because of incomplete clinical data (20 patients), were not receiving levodopa (33 patients), or had essential tremor characteristics in addition to parkinsonian features (2 patients). Further sub-classification of the BR population into moderate and severe presentations was also carried out. Moderate BR was defined as taking 51–100 mg of levodopa per dose, and severe BR was defined as taking 50 mg or less of levodopa per dose, and both sets of patients were required to manifest disabling dyskinesia. When measuring DBS outcomes in BR patients, data was obtained pre-operatively in the “on” medication state and 6 months post DBS surgery in the on medication state and on stimulation state.

### Statistical Analysis

Patient characteristics were compared using a Chi-square test corrected with an Exact test for the following variables: gender, ethnicity, smoker, family history, PD subtype, dopamine agonist, monoamine oxidase type B (MAO-B) inhibitor, amantadine, HY, early morning dystonia, and DBS; Mann-Whitney *U* test was used to assess for characteristics of dyskinesias such as waking day with dyskinesias, disability of dyskinesias, and painful dyskinesias; and Student’s *t* test for age, height, weight, body mass index (BMI), disease duration, milligrams of levodopa per dose, years on levodopa, levodopa equivalent dosage (LED), UPDRS scores, SE, BDI-II, and PDQ-39.

A total of 345 patients were analyzed, and *P* values<0.05 were considered statistically significant. Bonferroni-corrected p-values for multiple comparisons are shown in Table 1. Statistical analyses were performed using commercially available statistical software (SPSS, version 19.0; SPSS, Inc., Chicago, Illinois).

**Table 1:** Baseline and clinical characteristics of “non-brittle response” vs. “brittle response” patients.

	NBR (n = 326)	BR (n = 19)	*P* Value
Female, No. (%)	95 (29)	11 (58)	.008*
Age, mean (SD), y	68.1 (10.2)	63.4 (12.4)	.06
Caucasian, No. (%)	300 (92)	17 (90)	.39
Former/current smoker, No. (%)	99 (30)	8 (42)	.18
Family history of PD, No. (%)	26 (8)	4 (21)	.07
Height, mean (SD), cm	172.4 (10.8)	168.3 (9.3)	.11
Weight, mean (SD), kg	79.6 (18.5)	63.5 (16.7)	<.001*,**
Body mass index, mean (SD)	26.5 (4.9)	22.3 (4.8)	<.001*,**
Tremor-dominant PD, No. (%)	177 (54)	11 (58)	.72
Disease duration, mean (SD), y	8.9 (5.2)	12.6 (7.5)	.003*
Years on levodopa, mean (SD), y	5.9 (4.3)	9.8 (7.5)	.001*,**
Levodopa per dose, mean (SD), mg	180.2 (72.5)	78.3 (25.6)	<.001*,**
Dopamine agonist, No. (%)	109 (33)	11 (58)	.11
MAO-B inhibitor, No. (%)	72 (22)	3 (16)	.75
Amantadine, No. (%)	46 (14)	6 (32)	.05
LED, mean (SD)	895.1 (501.8)	601.6 (321.3)	.001*,**
UPDRS, mean (SD)			
Part I	2.9 (2.2)	2.8 (2.1)	.88
Part II	13.5 (6.7)	17.4 (6.0)	.03*
Part III	30.0 (12.9)	40.4 (16.4)	.001*,**
Part IV	3.5 (2.8)	7.3 (2.9)	<.001*,**
Total	50.5 (19.7)	65.1 (19.1)	.009*
HY I-III stage, No. (%)	300 (92.1)	16 (84)	.21
Schwab-England, mean (SD)	79.3 (17.2)	73 (26.7)	.48
BDI-II, mean (SD)	9.7 (78)	8.7 (4.9)	.62
PDQ-39, mean (SD)	213.8 (130.7)	250.1 (119.7)	.31
Currently on DBS, No. (%)	61 (18.7)	12 (63)	.001*

*significant at p<.05.

**significant at p<.002 (Bonferroni adjusted p-value for multiple comparisons).

BDI-II  =  Beck Depression Inventory; BR  =  brittle response; DBS  =  Deep Brain Stimulation; HY  =  Hoehn and Yahr; LED  =  levodopa equivalent dosage; MAO-B  =  monoamine oxidase type B; NBR  =  non-brittle response; PD  =  Parkinson’s disease; PDQ-39 =  Parkinson’s Disease Questionnaire; UPDRS  =  Unified Parkinson’s Disease Rating Scale.

## Results

### “Brittle Response” Population: General Characteristics

Of 345 patients, 19 (5.5%) met criteria for “brittle response” PD. The BR group was comprised predominantly of females (11/19), significantly different (*P* = .008) when compared to the NBR. The mean age of the BR population was 63.4±12.4 years. The BR group had significantly lower mean weight (63.5±16.7, *P = <*.001) and mean BMI (22.3±4.8, *P = <*.001) when compared to the NBR population. Eleven patients had tremor-dominant PD subtype. Another interesting finding was that the patients in the BR group had a significantly longer mean disease duration when compared to the NBR group (12.6±7.5 vs. 8.9±5.2 years, *P* = .003). These patients had been on LD therapy for an average duration of 9.8±7.5 years, which was also significantly longer when compared to the non-brittle response cohort (*P* = .001), who had been on LD therapy for only 5.9 4.3 years. Eleven patients in the BR group were also receiving a dopamine agonist, with 6 patients receiving amantadine and 3 patients on rasagiline. The mean LD per dose was 78.3±25.6 mg for the BR patients with a LED of 601.6±321.3. These dosages were significantly lower when compared to the NBR group (*P = <*.001 and *P* = .001, respectively).

The BR group had UPDRS motor and total scores of 40.4±16.4 and 65.1±19.1, respectively. According to HY staging, 16 patients had a stage rating between I-III, and the mean SE score in BR group was of 73±26.7. The BDI-II score of 8.7±4.9 revealed that these patients had minimal depressive symptoms, and were no different from the NBR group, and the PDQ-39 summary score of 250.1±119.7 revealed that the patients had mildly affected health-related quality of life profile. Table 1 describes demographic and clinical characteristics of both groups.

### “Brittle Response” Population: Characteristics of Dyskinesias

Since the goal of the study was to ascertain “brittle response” in PD and to describe those patients who were susceptible to adverse motor effects when treated with small amounts of levodopa, we assessed for the presence of dyskinesias using the UPDRS part IV items 32–35. Our analysis revealed that BR patients have far more dyskinesia during waking day than the NBR group (53% vs. 9% of patients experienced dyskinesia for more than 26% of waking hours); furthermore dyskinesias were rated as moderately to severely disabling in 11 of 19 patients, and 4 experienced painful dyskinesias. Ten patients in the BR population experienced early morning dystonia. Table 2 summarizes the characteristics of dyskinesia(s) in both populations.

**Table 2:** Characteristics of dyskinesias in “non-brittle response” vs. “brittle response” patients assessed by UPDRS part IV items 32–35.

	NBR (n = 95)	BR (n = 19)	*P* Value
>26% of waking day with dyskinesias, No. (%)	27 (9)	10 (53)	<.001
Moderate-severe disabling dyskinesias, No. (%)	11 (4)	11 (58)	<.001
Painful dyskinesias, No. (%)	8 (3)	4 (21)	<.001
Early morning dystonia, No. (%)	94 (29)	10 (53)	.06

BR  =  brittle response; NBR  =  non-brittle response.

### “Brittle Response” Population: Moderate vs. Severe

A secondary analysis of the BR patients was performed by dividing them into two groups: the moderate BR group for patients who required 51–100 mg of levodopa per dose with disabling dyskinesia; and a severe BR group for patients requiring 50 mg or less mg per dose; also with disabling dyskinesia. Only six patients met criteria for the “severe” group, and all were female. This group had a mean age of 67.7 years, and mean disease duration of 16.7 years. Furthermore, all patients in the group had a low BMI (mean 19.7) with 14.6 mean years on LD therapy. The severe BR group experienced more dyskinesia during the waking day, and the dyskinesia was rated as more severe than the moderate brittle group. Table 3 compares the “moderate” and the “severe” sub-groups.

**Table 3:** Comparison between subgroups of “brittle response” patients.

	Moderate (n = 13)	Severe (n = 6)
Female, No.	5	6
Age, mean (SD), y	61.5 (14.0)	67.7 (6.8)
Body mass index, mean (SD)	23.4 (5.1)	19.7 (2.9)
Disease duration, mean (SD), y	10.8 (3.9)	16.7 (10.0)
Years on levodopa, mean (SD), y	7.4 (4.5)	14.6 (10.5)
Total UPDRS, mean (SD)	65.9 (17.2)	62.3 (28.9)
Hoehn-Yahr stage, mean (SD)	2.8 (0.7)	2.8 (0.8)
>26% of waking day with dyskinesias, No.	6	4
Moderate-severe disabling dyskinesias, No.	7	4
Painful dyskinesias, No.	3	1
Early morning dystonia, No.	5	5
Currently on DBS, No.	7	5

DBS  =  Deep Brain Stimulation; UPDRS  =  Unified Parkinson’s Disease Rating Scale.

### “Brittle Response” Population: DBS Outcomes

Twelve of 19 BR patients were treated with DBS. Of this subgroup, 8 were female. The target nucleus for DBS therapy was variable across patients; 5 had bilateral subthalamic nucleus (STN), 2 had bilateral globus pallidus internus (GPi), and 5 had unilateral GPi. The mean pre-operative off and on meds UPDRS-III score was 44.3±15.8/29±12.1, with post-operative scores off meds/on stim 30±13.8 and on stim/on meds 28.2±15.6 at 6 months. Table 4 summarizes clinical outcomes in BR DBS patients. UPDRS motor scores were comparable to our sixty-one NBR DBS population of patients, whose scores were as follows: preoperative off/on meds 39.7±10.1/23.9±9.5, and postoperative off meds/on stim 28.9±9.4, on stim/on meds 20.1±7.9 at 6 months. We observed similar outcomes in UPDRS motor scores and dyskinesias in both DBS targets. We also observed an increase in weight in BR STN implanted patients (67.8 kg vs. 70.9 kg), however, GPi implanted patients kept the same weight (55.4 kg vs. 55.6 kg) at 6 months follow-up post-DBS.

**Table 4:** Clinical response to DBS in “brittle response” patients (n = 12).

	Baseline	6 months
UPDRS part III, mean (SD)	29 (12.1)	28.2 (15.6)
>26% of waking day with dyskinesias, No.	7	1
Moderate-severe disabling dyskinesias, No.	8	1
Early morning dystonia, No.	8	5
Mean (SD) Schwab and England	65 (35)	45.8 (46.2)
Mean (SD) BDI-II	10.2 (6.3)	5.1 (2.7)
Mean (SD) PDQ-39	286.1 (113.7)	228.0 (123.5)

BDI-II  =  Beck Depression Inventory; PDQ-39 =  Parkinson’s Disease Questionnaire; UPDRS  =  Unified Parkinson’s Disease Rating Scale.

## Discussion

The term “brittle” has been previously used in medicine to describe extreme blood sugar sensitivity in diabetics [2]. However, no formal definition has been proposed for the use of the term in the context of patients with PD, although it has been alluded to in a single experimental study [3]. Levodopa-induced dyskinesias (LID) could be produced by using a minimum dose of levodopa (0.66±0.08 mg/kg/hr.) in patients with PD [5] or alternatively by using a cumulative LD dose of 300 mg-years [6]. However, it should be kept in mind that many factors might impact the occurrence of LID. Many experts have anecdotally observed that dyskinesia may occur at less than 50 mg per dose. It is generally agreed that there is a direct relationship between LD dosage and induction of dyskinesia in many patients [7]. We were challenged in this project to develop a definition of a “brittle response” in patients with dyskinesia at very low doses. We arbitrarily chose as our working definition, 100 mg of levodopa per dose or less and patients were required to report symptoms of disabling dyskinesia(s) in their most recent visit. We believe that because of being the most commonly prescribed dose of levodopa, it allowed the capture and characterization of the phenomenon, excluding patients intolerant to medication or with gastrointestinal side effects. Motor fluctuations were not considered in the definition, as this complication was not the predominant issue for these patients who were all taking very low doses of levodopa. Because the aim was to emphasize the appearance of dyskinesia with low doses of medication this was the reason that levodopa per day was not analyzed. In our study, 5.5% of the total population met this definition. Patients in studies reporting frequency of dyskinesias and levodopa dosages, such as the ELLDOPA trial [7] and the STRIDE-PD study [8] were comprised of recently diagnosed PD (on levodopa for less than a year), rendering the groups not comparable.

The risk factors for BR patients uncovered in our cohort are consistent with recent studies describing risk factors for the occurrence of LID. The most significant factors found included female gender with low weight, as reported previously [1]. Duration of disease [9], levodopa dosage [7], [10], and duration of levodopa therapy [11] were also significant factors encountered. Although these factors may possibly be related to the greater degree of dopaminergic nigro-striatal depletion [12] and to a longer discontinuous or pulsatile stimulation of dopaminergic receptors [13], not all patients with long-standing PD seem to convert to “brittle response”, which imply this subgroup of patients may have specific characteristics which could place them at risk for maladaptive plastic responses [14]. Alternatively these patients may have a higher plasma and tissue concentration of levodopa in the setting of a low body weight [15], [16].

Although dyskinesias were in general not painful in either group, they were more frequent and more disabling in the BR patients, suggesting that this group could possibly have a greater sensitization to striatal dopamine, NMDA, and AMPA receptors [17], though this will need further clarification by future studies. Interestingly, no significant difference was observed in the Schwab and England scale, mood, or quality of life scales between populations. Even though being “brittle response” did not affect quality of life in this study, the differentiation of this subgroup may have pathophysiological and treatment implications.

Further descriptive analyses of the sub-groups revealed that “severe” BR patients were again female gender with low body weight. Again being consistent with previous literature describing female patients with low body weight [1] as risk factors for LID.

We observed that following DBS, BR patients had improvements in UPDRS motor, weight, BDI-II, and PDQ-39 scores. Dyskinesia improved in frequency and severity, thus revealing a beneficial effect of the surgical approach. Both the STN and GPi targets were effective for treatment. The on stim/on meds motor scores were slightly better than DBS preoperative on meds scores. A longer-term study with more subjects will be important to clarify if DBS in BR patients can provide more improvement in motor scores. The BR DBS patients likely had a more “benign” course of the disease (8 of 12 patients had tremor-dominant PD) and this may be a possible explanation for the longer disease duration, and the greater UPDRS scores.

We should take into consideration the following limitations of the study before generalizing our results to other PD populations. The study, though it did have a large sample size, was cross sectional and retrospective. Also, our sample may have been biased towards more severely affected patients, as it was comprised of PD patients who were referred to a tertiary specialist. The Dyskinesia Rating Scale [18] was not used to assess severity of dyskinesia, which would be ideal for future studies; instead we used UPDRS part IV item 33 in addition to PDQ-39 to overcome this limitation. Finally, a selection bias should be taken into consideration when considering results.

Female patients with a low body weight may have an increased risk for having a “brittle response” to PD medications. These patients are at higher risk for levodopa-induced dyskinesias and often require more complex pharmacological management, and thus will likely demand more resources from the healthcare system. DBS seems to work well for BR as comparable to NBR populations. It is not clear if the effect of weight gain [19], [20] as a result of DBS surgery will lead to less dyskinesia(s). It is also unknown what the best approach to treatment of the BR PD patient will be, and future studies should seek to expand on what is known about this phenotype.
